# Rates and Reasons for Early Change of First HAART in HIV-1-Infected Patients in 7 Sites throughout the Caribbean and Latin America

**DOI:** 10.1371/journal.pone.0010490

**Published:** 2010-06-01

**Authors:** Carina Cesar, Bryan E. Shepherd, Alejandro J. Krolewiecki, Valeria I. Fink, Mauro Schechter, Suely H. Tuboi, Marcelo Wolff, Jean W. Pape, Paul Leger, Denis Padgett, Juan Sierra Madero, Eduardo Gotuzzo, Omar Sued, Catherine C. McGowan, Daniel R. Masys, Pedro E. Cahn, for The Caribbean, Central and South America Network for HIV Research (CCASAnet) Collaboration, of the International Epidemiologic Databases to Evaluate AIDS (IeDEA) Program

**Affiliations:** 1 Fundación Huésped, Buenos Aires, Argentina; 2 Vanderbilt University School of Medicine, Nashville, Tennessee, United States of America; 3 Projeto Praça Onze, Hospital Universitário Clementino Fraga Filho and Universidade Federal do Rio de Janeiro, Rio de Janeiro, Brazil; 4 Fundación Arriarán and Facultad de Medicina, Universidad de Chile, Santiago, Chile; 5 Le Groupe Haïtien d'Etude du Sarcome de Kaposi et des Infections Opportunistes in Port-au-Prince (GHESKIO), Port-au-Prince, Haiti; 6 Instituto Hondureño de Seguridad Social and Universidad Autónoma de Honduras, Tegucigalpa, Honduras; 7 Instituto Nacional de Ciencias Médicas y Nutrición Salvador Zubirán, México City, México; 8 Universidad Peruana Cayetano Heredia Facultad de Medicina and Instituto de Medicina Tropical Alexander von Humboldt, Lima, Perú; University of Cape Town, South Africa

## Abstract

**Background:**

HAART rollout in Latin America and the Caribbean has increased from approximately 210,000 in 2003 to 390,000 patients in 2007, covering 62% (51%–70%) of eligible patients, with considerable variation among countries. No multi-cohort study has examined rates of and reasons for change of initial HAART in this region.

**Methodology:**

Antiretroviral-naïve patients > = 18 years who started HAART between 1996 and 2007 and had at least one follow-up visit from sites in Argentina, Brazil, Chile, Haiti, Honduras, Mexico and Peru were included. Time from HAART initiation to change (stopping or switching any antiretrovirals) was estimated using Kaplan-Meier techniques. Cox proportional hazards modeled the associations between change and demographics, initial regimen, baseline CD4 count, and clinical stage.

**Principal Findings:**

Of 5026 HIV-infected patients, 35% were female, median age at HAART initiation was 37 years (interquartile range [IQR], 31–44), and median CD4 count was 105 cells/uL (IQR, 38–200). Estimated probabilities of changing within 3 months and one year of HAART initiation were 16% (95% confidence interval (CI) 15–17%) and 28% (95% CI 27–29%), respectively. Efavirenz-based regimens and no clinical AIDS at HAART initiation were associated with lower risk of change (hazard ratio (HR) = 1.7 (95% CI 1.1–2.6) and 2.1 (95% CI 1.7–2.5) comparing neverapine-based regimens and other regimens to efavirenz, respectively; HR = 1.3 (95% CI 1.1–1.5) for clinical AIDS at HAART initiation). The primary reason for change among HAART initiators were adverse events (14%), death (5.7%) and failure (1.3%) with specific toxicities varying among sites. After change, most patients remained in first line regimens.

**Conclusions:**

Adverse events were the leading cause for changing initial HAART. Predictors for change due to any reason were AIDS at baseline and the use of a non-efavirenz containing regimen. Differences between participant sites were observed and require further investigation.

## Introduction

An estimated 1.93 million people live with HIV in Latin America and the Caribbean, comprising 5.7% of all infected persons worldwide; the adult prevalence in this region is 0.5%[1]. Access to antiretroviral (ARV) therapy has improved and at the end of 2007 approximately 390,000 patients in this region were receiving antiretroviral therapy with an overall coverage of 62% (51%–70%), although considerable variation exists between countries[2], [3]. Unfortunately, 75% of patients still initiate treatment at advanced stages of disease[4]–[8].

Treatment toxicities and adherence problems may lead to suboptimal therapy, discontinuation, and treatment failure. Early modification of initial highly active antiretroviral therapy (HAART) has been associated with poor clinical outcomes[9]. Therefore, knowing why patients modify therapy could improve our understanding of successful HAART, guide decisions regarding initiation and management of HAART in specific patient populations, and inform interventions to reduce HAART discontinuation.

The frequency and reasons for HAART change have been assessed by cohort studies from resource-rich and -limited settings, but Latin America and the Caribbean have been largely underrepresented in these studies[10]–[16]. Observational studies from sites in Argentina, Brazil, Haiti and Peru have described the occurrence of adverse events and durability of first regimen[17]–[23]. However, no multisite study has addressed frequency and reasons for change in this region.

The Caribbean, Central and South America Network for HIV Research (CCASAnet) collaboration includes sites from seven nations: Argentina, Brazil, Chile, Haiti, Honduras, Mexico, and Peru. In an earlier study of antiretroviral-naïve subjects starting HAART, mortality rates in the CCASAnet cohort were similar to those reported for resource-limited settings with a 1-year probability of death for the combined cohort of 8.3%, although this varied considerably across sites[8]. The purpose of the current study is to explore the frequency of, risk factors for, and reasons for changing/discontinuing HAART during the first year after initiation in the CCASAnet region.

## Methods

### Ethics Statement

This study was conducted according to the principles expressed in the Declaration of Helsinki. Institutional Review Board approval was obtained locally for each participating site and the coordinating centre: Comité de Bioética de Fundación Huésped; Comitê de Ética em Pesquisa-Universidade Federal Do Río De Janeiro; Comité Ético-Científico del Servicio de Salud Metropolitano Central, Ministerio de Salud, Gobierno de Chile; Human Research Protections Programs, Division of Research Integrity, Weill Cornell Medical College; Comité de Ética en Investigación Biomédica de la Unidad de Investigación Científica, Facultad de Ciencias Médicas, Universidad Nacional Autónoma de Honduras; Comité Institucional de Investigación Biomédica en Humanos, Instituto Nacional de Ciencias Médicas y Nutrición Salvador Zubirán; Vicerrectorado de Investigación, Dirección Universitaria de Investigación, Ciencia y Tecnología-DUICT, Universidad Peruana Cayetano Heredia; Institutional Review Board, Vanderbilt University.

All data were de-identified prior to being transmitted to the Vanderbilt Data Coordinating Centre.

In each of the countries contributing data to this study, ethical regulations and policies permit retrospective analysis of de-identified clinical data without informed consent when the research is approved by an appropriately constituted ethics committee or Institutional Review Board. These approvals were obtained in all cases and the need to obtain informed consent was waived by all of the ethics committees of the participating sites.

### Participants and Settings

The CCASAnet cohort (www.ccasanet.vanderbilt.edu) has been described elsewhere[24]. Briefly, the collaboration was established in 2006 as Region 2 of the International Epidemiologic Databases to Evaluate AIDS (IeDEA; www.iedea-hiv.org). The cohort includes 7 sites: Fundación Huésped in Buenos Aires, Argentina (FH-Argentina); Hospital Universitário Clementino Fraga Filho in Rio de Janeiro, Brazil (HUCFF-Brazil); Fundación Arriarán in Santiago, Chile (FA-Chile); Le Groupe Haïtien d'Etude du Sarcome de Kaposi et des Infections Opportunistes in Port-au-Prince, Haiti (GHESKIO-Haiti); Instituto Hondureño de Seguridad Social Hospital de Especialidades and Hospital Escuela in Tegucigalpa, Honduras (IHSS/HE-Honduras); Instituto Nacional de Ciencias Médicas y Nutrición Salvador Zubirán in Mexico City, Mexico (INNSZ-México); and Instituto de Medicina Tropical Alexander Von Humboldt in Lima, Peru (IMTAvH-Peru). Each cohort was established at a different time between 1996 and 2002 not necessarily reflecting the availability of HAART in each country.

Data audits were performed at each site by a team from the VDCC. The present analysis used data for the first year of follow-up after starting HAART collected through June 2008. Included were antiretroviral-naïve HIV-infected patients prescribed HAART at age 18 years or older with at least one follow-up visit.

Initiation of HAART at each site followed either national or World Health Organization guidelines[25]–[30]. Guidelines from Argentina, Brazil, Chile, Honduras and Mexico recommend drug substitutions after toxicity and switching regimens after virologic failure[25], [27]–[29]. In contrast, in Haiti and Peru failure was defined according to WHO clinical and immunologic criteria[30]. Table 1 lists site-specific practices related to initiation criteria, laboratory monitoring, and regimen availability.

**Table 1:** Treatment Program Characteristics across Sites.

Site	FH-Argentina	HUCFF-Brazil	FA-Chile	GHESKIO-Haiti	IHSS/HE-Honduras	INNSZ-Mexico	IMTAvH-Peru
Year of HAART expanded access	2000	1996	Late 2001	February 2003	July 2003	2001	May 2004
Funding source for ART	MOH	MOH	PHS, GFATM	GFATM, PEPFAR, NIH	MOH	MOHS	MOH, GFATM
Patients receiving free ART (%)	100	100	80	100	100	100	100
Generic ART use (%)	NRTI except TDF, NVP, EFV, SQV, RTV are generic	NRTIs, NVP, EFV, SQV, RTV are generic	NRTI except TDF, and NVP are generic	80	90	0	90
Guidelines used for ART Initiation and Change	National	National	National	WHO	National	National	WHO
Initial HAART regimens used to treat ≥95% of patients, n	21	21	9	5	2	19	9
AE reporting	Not standardized	Not standardized	Not standardized	Not standardized	Not standardized	Not standardized	Not standardized
Frequency of clinical visits							
At HAART initiation	2–4 weeks	2–4 weeks	2–4 weeks	2–4 weeks	2–4 weeks	3–6 weeks	1–2 weeks
On ongoing HAART	3–4 months	3–4 months	4–6 months	1 month	2-6 months	4 months	2-3 months
Use of virologic monitoring	Yes	Yes	Yes	No	Yes	Yes	Yes
Frequency of pharmacy pick-ups	1 month	1 month	1 month	1 month	1 month	1-2 months	1 month

ART, antiretrovial therapy; ARV, antiretroviral drug; MOH, Ministry of Health; GFATM, The Global Fund to Fight AIDS, Tuberculosis and Malaria; PEPFAR, US President's Emergency Plan for AIDS Relief; NIH, US National Institutes of Health

### Outcomes

The primary outcome was first change of regimen during the first year of HAART. Regimen change was defined as any alteration–switch or discontinuation–of ≥1 antiretroviral. Discontinuation was defined as simultaneous stopping of all antiretrovirals without initiation of a subsequent regimen for more than 30 days. Dosage adjustments and interruptions of therapy shorter than 30 days were ignored because of inconsistent recording of short interruptions across sites. Reasons for change were collected by each site and classified at the coordinating centre. Specific definitions of reasons for regimen change, including definition of treatment failure, were not standardized across sites and only included if they prompted a regimen change. Secondary analyses classified patients who died or were lost to follow-up (LTFU) while on their first HAART as having discontinued treatment. Patients without a visit for 6 months were classified as LTFU. The 6-month interval was chosen to include the longest interval between regular visits in participant sites, although most sites scheduled visits every 3 months.

### Data Sources and Measurements

Baseline CD4 count was defined as the measurement closest to HAART initiation but not more than 6 months prior to, or 7 days after, the date of HAART start. Baseline HIV-1 plasma viral load (PVL) was defined as the pre-HAART measurement closest to, but not more than 6 months prior to, HAART initiation. Baseline weight and hemoglobin were defined as the measurements closest to HAART initiation within +/− 30 days. HAART was defined as protease inhibitor (PI)-based (1 ritonavir-boosted or unboosted PI plus ≥2 nucleoside reverse-transcriptase inhibitors [NRTI]), non-nucleoside reverse transcriptase (NNRTI)-based (1 NNRTI plus ≥2 NRTIs), or other combinations (including triple NRTI regimens and any other regimen containing a minimum of three drugs). Clinical stage of disease was defined as AIDS (WHO stage 4, CDC stage C, or 1986 CDC stage 4), non-AIDS, or unknown.

### Statistical Analysis

Kaplan-Meier estimates computed probabilities of change per site. Time was measured from the start of HAART and ended at the earliest of regimen change, discontinuation, death, last visit before LTFU, last visit before the database closing, or 365 days. The closing date was defined separately for each site as the date of the most recent visit recorded in the database, and ranged from March 2007 to June 2008. The relationship between time to change and baseline variables was assessed using Cox proportional hazards models applied separately for each site. The primary multivariable analyses only included baseline predictors whose hazard ratio could be computed for all sites. Secondary, site-specific multivariable analyses included other routinely collected predictors with >50% non-missing data. In multivariable analyses, missing values of baseline predictors were accounted for using multiple imputation techniques applied separately within each site[31]. CD4 count and date of HAART initiation were included in models as continuous variables and expanded using restricted cubic splines to avoid linearity assumptions[32]. The combined hazard ratios and 95% confidence intervals (CI) were computed based on the results of site-specific hazard ratios using the meta-analysis approach of DerSimonian and Laird [33], a random effects method which makes no assumption regarding proportional hazards across sites[34]. All analyses were performed using R statistical software, version 2.8.1 (http://www.r-project.org). Analysis scripts are available at http://ccasanet.vanderbilt.edu/files/public/switch.nw

## Results

A total of 5026 naïve patients starting HAART with at least one follow-up visit were included. Patient characteristics at HAART initiation are summarized by site in Table 2. Across all sites, 35% were female, median age was 37 years, median CD4 count was 105 cells/µL (interquartile range [IQR]: 38, 200), 47% of subjects had clinical AIDS, and 78% of subjects had either CD4<200 cells/µL or clinical AIDS.

**Table 2:** Summary of Patient Characteristics, Calendar Year, and Regimens at HAART Initiation.

	FH-Argentina	HUCFF-Brazil	FA-Chile	GHESKIO-Haiti	IHSS/HE-Honduras	INNSZ-Mexico	IMTAvH-Peru	Combined
	n = 720 (14%)	n = 522 (10%)	n = 546 (11%)	n = 1646 (33%)	n = 324 (6%)	n = 414 (8%)	n = 854 (17%)	n = 5026
Female	209 (29%)	175 (33.5%)	69 (12.6%)	879 (53.4%)	128 (39.5%)	52 (12.6%)	253 (29.6%)	1765 (35.1%)
Age a	36 (31, 43)	37 (32, 45)	36 (30, 42)	39 (33, 45)	37 (31, 41)	35 (29, 42)	34 (28, 40)	37 (31, 44)
Route of Infection								
Heterosexual b	249 (47.3%)	219 (62.2%)	160 (29.6%)	-	323 (99.7%) c	148 (35.7%)	583 (68.8%)	1682 (56%) c
IVDU	66 (12.5%)	7 (2%)	2 (0.4%)	-	1 (0.3%)	0 (0%)	0 (0%)	76 (2.5%)
MSM	208 (39.5%)	106 (30.1%)	378 (70%)	-	0 (0%)	263 (63.5%)	264 (31.1%)	1219 (40.6%)
Other	3 (0.6%)	20 (5.7%)	0 (0%)	-	0 (0%)	3 (0.7%)	1 (0.1%)	27 (0.9%)
Missing	194 (26.9%)	170 (32.6%)	6 (1.1%)	1646 (100%)	0 (0%)	0 (0%)	6 (0.7%)	2022 (40.2%)
Clinical Stage								
not AIDS b	487 (68%)	95 (26.2%)	302 (55.4%)	944 (57.4%)	186 (59.2%)	226 (56.2%)	305 (37.3%)	2545 (53%)
AIDS	229 (32%)	267 (73.8%) d	243 (44.6%)	702 (42.6%)	128 (40.8%)	176 (43.8%)	512 (62.7%)	2257 (47%)
Missing	4 (0.4%)	160 (25.4%)	1 (0.1%)	0 (0%)	10 (2.3%)	12 (2.1%)	37 (2.8%)	224 (4.7%)
CD4 (cells/mL) a	155 (52, 245)	153 (53, 240)	116 (32, 193)	102 (37, 192)	105 (55, 185)	88 (33, 193)	79 (32, 164)	105 (38, 200)
<50 b	150 (24.2%)	80 (24.1%)	142 (32.3%)	434 (30.4%)	49 (19.1%)	136 (34.3%)	254 (36.9%)	1245 (29.9%)
50-99	85 (13.7%)	51 (15.4%)	65 (14.8%)	258 (18.1%)	75 (29.3%)	74 (18.7%)	145 (21.1%)	753 (18.1%)
100-199	163 (26.2%)	78 (23.5%)	133 (30.3%)	407 (28.5%)	77 (30.1%)	95 (24%)	165 (24%)	1118 (26.9%)
200-349	156 (25.1%)	102 (30.7%)	91 (20.7%)	283 (19.8%)	47 (18.4%)	84 (21.2%)	99 (14.4%)	862 (20.7%)
≥350	67 (10.8%)	21 (6.3%)	8 (1.8%)	47 (3.3%)	8 (3.1%)	7 (1.8%)	25 (3.6%)	183 (4.4%)
Missing e	99 (13.8%)	190 (36.4%)	107 (19.6%)	217 (13.2%)	68 (21%)	18 (4.3%)	166 (19.4%)	865 (17.2%)
CD4<200 or AIDS								
No b	255 (35.5%)	116 (25.1%)	96 (17.6%)	360 (21.9%)	72 (22.8%)	74 (17.9%)	127 (15.1%)	1100 (22.2%)
Yes	463 (64.5%)	347 (74.9%)	449 (82.4%)	1286 (78.1%)	244 (77.2%)	339 (82.1%)	716 (84.9%)	3844 (77.8%)
Missing	2 (0.3%)	59 (11.3%)	1 (0.2%)	0 (0%)	8 (2.5%)	1 (0.2%)	11 (1.3%)	82 (1.6%)
HIV-1 RNA (log_10_) a	5 (4.5, 5.4)	4.7 (3.6, 5.2)	5.1 (4.7, 5.5)	-	5 (4.7, 5)	4.9 (4.9, 4.9)	5.2 (4.7, 5.5)	5 (4.6, 5.4)
<10,000 b	13 (2.6%)	8 (5.3%)	1 (0.3%)	-	2 (2.2%)	0 (0%)	33 (6.8%)	57 (2.9%)
10,000-99,999	20 (4%)	23 (15.2%)	1 (0.3%)	-	4 (4.3%)	3 (0.8%)	0 (0%)	51 (2.6%)
≥100,000	470 (93.4%)	120 (79.5%)	383 (99.5%)	-	87 (93.5%)	353 (99.2%)	451 (93.2%)	1864 (94.5%)
Missing	217 (30.1%)	371 (71.1%)	161 (29.5%)	1646 (100%)	231 (71.3%)	58 (14%)	370 (43.3%)	3054 (60.8%)
Weight (kg) a	66 (60, 74)	65 (58, 74)	64 (56, 72)	53 (47, 60)	59 (51, 65)	61 (53, 71)	57 (50, 64)	56 (49, 64)
Missing	647 (89.9%)	399 (76.4%)	325 (59.5%)	92 (5.6%)	94 (29%)	105 (25.4%)	212 (24.8%)	1874 (37.3%)
Hemoglobin (g/dL) a	13.8 (11.8, 14.6)	11.7 (10, 13.7)	12.3 (10.7, 13.7)	10 (9, 11)	12.3 (10.8, 13.5)	14.2 (11.8, 15.7)	11.3 (10.3, 12.7)	11 (9.9, 13)
Missing	697 (96.8%)	267 (51.1%)	343 (62.8%)	528 (32.1%)	143 (44.1%)	165 (39.9%)	683 (80%)	2826 (56.2%)
Calendar Year								
1996-1999	6 (0.8%)	136 (26.1%)	0 (0%)	0 (0%)	3 (0.9%)	0 (0%)	5 (0.6%)	150 (3%)
2000-2001	42 (5.8%)	124 (23.8%)	35 (6.4%)	0 (0%)	5 (1.5%)	3 (0.7%)	11 (1.3%)	220 (4.4%)
2002-2003	210 (29.2%)	118 (22.6%)	290 (53.1%)	699 (42.5%)	104 (32.1%)	147 (35.5%)	50 (5.9%)	1618 (32.2%)
2004-2005	325 (45.1%)	80 (15.3%)	221 (40.5%)	923 (56.1%)	100 (30.9%)	161 (38.9%)	520 (60.9%)	2330 (46.4%)
2006-2007	137 (19%)	64 (12.3%)	0 (0%)	24 (1.5%)	112 (34.6%)	103 (24.9%)	268 (31.4%)	708 (14.1%)
Initial Regimen								
NNRTI	469 (65.1%)	288 (55.2%)	510 (93.4%)	1569 (95.3%)	311 (96%)	287 (69.3%)	788 (92.3%)	4222 (84%)
EFV-based	316 (67.4%)	238 (82.6%)	326 (63.9%)	922 (58.8%)	220 (70.7%)	271 (94.4%)	176 (22.3%)	2469 (58.5%)
NVP-based	153 (32.6%)	50 (17.4%)	184 (36.1%)	647 (41.2%)	91 (29.3%)	16 (5.6%)	612 (77.7%)	1753 (41.5%)
PI	21 (2.9%)	167 (32%)	23 (4.2%)	15 (0.9%)	10 (3.1%)	16 (3.9%)	21 (2.5%)	273 (5.4%)
Boosted PI	188 (26.1%)	43 (8.2%)	4 (0.7%)	7 (0.4%)	2 (0.6%)	106 (25.6%)	36 (4.2%)	386 (7.7%)
Other	42 (5.8%)	24 (4.6%)	9 (1.6%)	55 (3.3%)	1 (0.3%)	5 (1.2%)	9 (1.1%)	145 (2.9%)
ZDV Containing	542 (75.3%)	422 (80.8%)	432 (79.1%)	1515 (92%)	229 (70.7%)	289 (69.8%)	631 (73.9%)	4060 (80.8%)
Most Common Regimens								
3TC,ZDV,EFV	249 (34.6%)	196 (37.5%)	237 (43.4%)	855 (51.9%)	218 (67.3%)	194 (46.9%)	121 (14.2%)	2070 (41.2%)
3TC,ZDV,NVP	130 (18.1%)	37 (7.1%)	167 (30.6%)	593 (36%)	1 (0.3%)	9 (2.2%)	475 (55.6%)	1412 (28.1%)
3TC,D4T,NVP	19 (2.6%)	6 (1.1%)	6 (1.1%)	49 (3%)	90 (27.8%)	2 (0.5%)	95 (11.1%)	267 (5.3%)
3TC,D4T,EFV	51 (7.1%)	29 (5.6%)	55 (10.1%)	55 (3.3%)	0 (0%)	21 (5.1%)	23 (2.7%)	234 (4.7%)
3TC,ABC,ZDV	32 (4.4%)	22 (4.2%)	8 (1.5%)	55 (3.3%)	0 (0%)	4 (1%)	2 (0.2%)	123 (2.4%)

a Continuous variables are reported as medians (interquartile range).

b Percentages are computed using the number of patients with a non-missing value.

c IHSS/HE-Honduras did not differentiate between heterosexual and MSM routes of infection. All are listed here as heterosexual.

d HUCFF-Brazil classified most patients with CD4<200 as having clinical AIDS.

e Sites may have high percentages of missing CD4, HIV-1 RNA, weight, and hemoglobin values because data were not collected within the specified time frames to count as baseline measurements (see Methods: Data Sources and Measurements).

Table 2 also describes initial HAART regimen per site. The majority started HAART between 2002–2005 although 26% of patients from HUCFF-Brazil initiated prior to 2000. Across sites, NNRTI-based initial regimens were most common (84%) with efavirenz (EFV) the most frequently used (58.5%) except in IMTAvH-Peru. Eight percent of initial regimens were ritonavir-boosted PI-based: saquinavir (34%), lopinavir (31%) and indinavir (26%). Unboosted PI-based regimens accounted for 5% of initial regimens in the combined cohort; but were commonly used before 2000 in HUCFF-Brazil. Other regimens were mainly triple NRTIs (89%). Among nucleosides, lamivudine (3TC) was included in nearly all initial regimens (97%). Zidovudine (ZDV) was included in nearly 80% of all initial regimens; 84% and 81% of patients on EFV- and NVP-based regimens, respectively, were on ZDV, compared to 70% of patients who were not started on NNRTI-based regimens. Seventy percent of regimens which did not contain ZDV contained d4T. Didanosine and abacavir were used in only 4.3% and 4.6% of overall regimens, respectively, and tenofovir was used rarely (1.7%). The most common initial regimens were 3TC, ZDV, EFV (41.2%); 3TC, ZDV, NVP (28.1%); 3TC, d4T, NVP (5.3%); and 3TC, d4T, EFV (4.7%).

**Figure 1:**
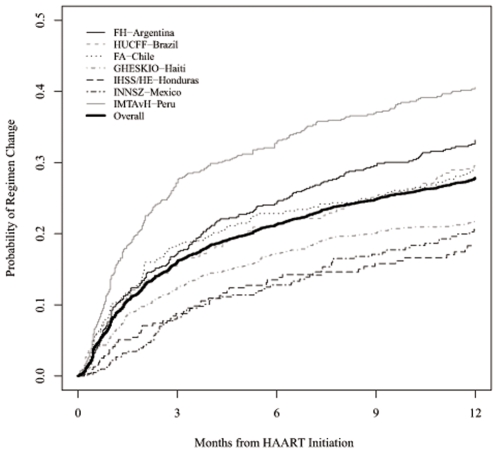
Probability of regimen change by site.

Figure 1 shows Kaplan-Meier estimates of the probability of changing/discontinuing regimens during the first year by site. The estimated 3-month and 1-year probabilities of change (95% CI) for the combined cohort were 16% (15–17%) and 28% (27–29%) respectively (Table 3). Regimen change during the first year was lowest at IHSS/HE-Honduras and highest at IMTAvH-Peru. Two -hundred eighty-six patients (5.7%) died during the first year prior to changing regimens and 149 patients (3.0%) were LTFU. When these patients were analyzed as having discontinued regimens, then the estimated 3-month and 1-year probabilities of change/discontinuation were 21% (95% CI 20–22%) and 36% (95% CI 34–37%), respectively.

**Table 3:** Number of Events and Estimated Rates of Changing Regimens (95% Confidence Intervals).

	FH-Argentina	HUCFF-Brazil	FA-Chile	GHESKIO-Haiti	IHSS/HE-Honduras	INNSZ-Mexico	IMTAvH-Peru	Combined
	(n = 720)	(n = 522)	(n = 546)	(n = 1646)	(n = 324)	(n = 414)	(n = 854)	(n = 5026)
1 year Change/Discontinuation	223 (30.9%)	151 (28.9%)	154 (28.2%)	324 (19.7%)	52 (16.0%)	77 (18.65)	307 (35.9%)	1288 (25.6%)
Single Drug Substitution	82 (11.4%)	77 (14.8%)	94 (17.2%)	294 (17.9%)	22 (6.8%)	35 (8.5%)	156 (18.3%)	760 (15.1%)
Other Regimen Change	64 (8.9%)	45 (8.6%)	11 (2.0%)	29 (1.8%)	29 (9.0%)	40 (9.7%)	70 (8.2%)	288 (5.7%)
Discontinuation	77 (10.7%)	29 (5.5%)	49 (9.0%)	1 (0.1%)	1 (0.3%)	2 (0.5%)	81 (9.5%)	240 (4.8%)
Death	6 (0.8%)	7 (1.3%)	18 (3.3%)	169 (10.3%)	30 (9.3%)	11 (2.7%)	45 (5.3%)	286 (5.7%)
Loss to Follow-up (LTFU)	52 (7.2%)	10 (1.9%)	6 (1.1%)	44 (2.7%)	1 (0.3%)	15 (3.6%)	21 (2.5%)	149 (3.0%)
Rates of Change/Discontinuation								
3 months	0.17 (0.15, 0.2)	0.16 (0.13, 0.19)	0.18 (0.15, 0.21)	0.13 (0.11, 0.14)	0.09 (0.06, 0.12)	0.08 (0.06, 0.11)	0.28 (0.25, 0.31)	0.16 (0.15, 0.17)
1 year	0.33 (0.29, 0.37)	0.3 (0.25, 0.33)	0.29 (0.25, 0.33)	0.22 (0.2, 0.24)	0.18 (0.14, 0.23)	0.21 (0.17, 0.25)	0.41 (0.37, 0.44)	0.28 (0.27, 0.29)
Including death								
3 months	0.18 (0.15, 0.21)	0.17 (0.14, 0.2)	0.2 (0.17, 0.24)	0.19 (0.17, 0.21)	0.15 (0.11, 0.19)	0.09 (0.06, 0.12)	0.31 (0.28, 0.34)	0.2 (0.19, 0.21)
1 year	0.34 (0.3, 0.37)	0.31 (0.27, 0.35)	0.32 (0.28, 0.36)	0.31 (0.28, 0.33)	0.27 (0.22, 0.32)	0.24 (0.19, 0.28)	0.45 (0.41, 0.48)	0.33 (0.31, 0.34)
Including LTFU and death								
3 months	0.21 (0.18, 0.24)	0.17 (0.14, 0.21)	0.21 (0.17, 0.24)	0.21 (0.19, 0.23)	0.15 (0.11, 0.19)	0.1 (0.07, 0.13)	0.32 (0.29, 0.35)	0.21 (0.2, 0.22)
1 year	0.4 (0.36, 0.44)	0.32 (0.28, 0.36)	0.33 (0.29, 0.37)	0.33 (0.31, 0.35)	0.27 (0.22, 0.32)	0.27 (0.23, 0.32)	0.47 (0.43, 0.51)	0.35 (0.34, 0.37)

Most regimen changes were simple drug substitutions followed by other regimen changes and discontinuations.

Of 1288 living patients whose initial HAART was changed, 1147 (89%) switched to a different regimen, 104 (8%) did not start a second regimen during the observed follow-up, and 37 (3%) re-started their initial regimen after an interruption ≥30 days. For those patients who re-started the same regimen during the first year, the median time of interruption was 78 days. For those who switched to a different regimen, the vast majority (83%) started within a week of initial HAART discontinuation and 90% within a month. Among those who started a second regimen while in follow-up, 74% were NNRTI-based and 5% were first line PI-based. Of 1013 living patients who stopped their initial ZDV-containing regimen, 487 had a second regimen containing ZDV, 452 had a second regimen not containing ZDV, and 74 did not start a second regimen. Table 4 details second regimens started within 30 days of changing initial regimen.

**Table 4:** Second Regimens started within 30 days of stopping Initial Regimen.

	Initial Regimen					
	NNRTI-EFV (n = 2469)	NNRTI-NVP (n = 1753)	Boosted PI (n = 386)	PI (n = 273)	Other (n = 145)	Total (n = 5026)
None, Death	146 (5.9%)	118 (6.7%)	13 (3.4%)	4 (1.5%)	5 (3.4%)	286 (5.7%)
None a	68 (2.8%)	111 (6.3%)	33 (8.5%)	18 (6.6%)	10 (6.9%)	240 (4.8%)
NNRTI-EFV	200 (8.1%)	109 (6.2%)	39 (10.1%)	12 (4.4%)	52 (35.9%)	412 (8.2%)
NNRTI-NVP	166 (6.7%)	176 (10.0%)	8 (2.1%)	11 (4%)	7 (4.8%)	368 (7.3%)
Boosted PI b	29 (1.2%)	35 (2.0%)	34 (8.8%)	13 (4.8%)	7 (4.8%)	118 (2.3%)
Unboosted PI	30 (1.2%)	19 (1.1%)	6 (1.6%)	34 (12.5%)	2 (1.4%)	91 (1.8%)
Other	9 (0.4%)	9 (0.6%)	6 (1.6%)	3 (1.1%)	2 (1.4%)	29 (0.6%)
Non-HAART Regimen	1 (0%)	25 (1.4%)	0 (0%)	4 (1.5%)	0 (0%)	30 (0.6%)

a Of the 240 living patients who did not start a second regimen within 30 days of stopping their first regimen, 136 (56.7%) started a second regimen before the end of follow-up.

b In the second regimen there were 54 first generation boosted PIs (SQV, IDV, NFV, or APV) and 93 second generation boosted PIs (LPV, FPV, ATV, DRV or TPV)

Clinical AIDS at HAART initiation and non-efavirenz based regimens were associated with a higher hazard of change in unadjusted analyses for most sites and in the combined cohort (data not shown). Multivariable analyses for each site and pooled across sites are given in Table 5. After adjusting for sex, age, baseline CD4 count, year of HAART initiation, and type of regimen, the hazard of change was 1.3 times higher for a person with clinical AIDS prior to HAART initiation than a person without (95% CI: 1.1 to 1.5) (Table 3). Using EFV-based regimens as the reference category, the hazard ratios for change for NVP-based regimens and non-NNRTI-based regimens were 1.7 (95% CI: 1.1 to 2.6) and 2.1 (95% CI: 1.7 to 2.5) respectively. The increased hazard for change for NVP was not observed in FA-Chile and GHESKIO-Haiti whereas it was especially pronounced in IMTAvH-Peru. Except at GHESKIO-Haiti, patients who started 3TC,ZDV,EFV generally had lower rates of change than those starting other regimens (Table S1, online supplemental material). Overall and by site there were no consistent associations between gender, age, CD4, year of HAART initiation, or ZDV-containing regimens and change. Results were similar when those who died and those who died or were lost to follow-up were assumed to have discontinued regimens (Tables S2-S3, online supplemental material).

**Table 5:** Adjusted Hazard Ratios (95% Confidence Intervals) for Regimen Change/Discontinuation in First Year.

	FH-Argentina (n = 720)	HUCFF-Brazil (n = 522)	FA-Chile (n = 546)	GHESKIO-Haiti (n = 1646)	IHSS/HE-Honduras (n = 324)	INNSZ-Mexico (n = 414)	IMTAvH-Peru (n = 854)	Combined
Male	0.73 (0.55, 0.98)	1.07 (0.75, 1.52)	0.78 (0.49, 1.23)	0.76 (0.58, 0.99)	0.97 (0.55, 1.72)	0.97 (0.5, 1.92)	1.06 (0.82, 1.38)	0.89 (0.78, 1.02)
Age (per 10 years)	1.02 (0.89, 1.18)	1 (0.85, 1.17)	0.96 (0.81, 1.14)	0.93 (0.82, 1.05)	1 (0.73, 1.37)	0.88 (0.69, 1.12)	0.97 (0.86, 1.09)	0.97 (0.91, 1.03)
AIDS	1.61 (1.18, 2.2)	1.37 (0.89, 2.11)	1.18 (0.79, 1.75)	1.13 (0.89, 1.42)	0.87 (0.49, 1.57)	1.42 (0.83, 2.44)	1.26 (0.98, 1.63)	1.28 (1.14, 1.45)
CD4 count (cells/mL)								
100 vs. 50	1.13 (1.05, 1.21)	0.9 (0.82, 0.99)	1.02 (0.91, 1.14)	0.97 (0.91, 1.04)	0.98 (0.8, 1.2)	0.92 (0.78, 1.08)	0.9 (0.84, 0.97)	0.98 (0.91, 1.05)
200 vs. 50	1.33 (1.12, 1.59)	0.78 (0.62, 0.98)	1.04 (0.79, 1.39)	0.94 (0.79, 1.1)	0.94 (0.58, 1.54)	0.81 (0.55, 1.21)	0.78 (0.66, 0.93)	0.94 (0.79, 1.13)
350 vs. 50	1.6 (1.2, 2.14)	0.66 (0.45, 0.96)	1.07 (0.67, 1.71)	0.9 (0.68, 1.17)	0.91 (0.41, 2.03)	0.71 (0.37, 1.36)	0.67 (0.5, 0.89)	0.91 (0.67, 1.22)
Year of HAART initiation								
2003 (ref) a	1	1	1	1	1	1	1	1
2004	1.06 (0.96, 1.18)	1.12 (1, 1.26)	0.99 (0.83, 1.16)	0.35 (0.27, 0.44)	0.94 (0.75, 1.18)	0.94 (0.75, 1.19)	0.99 (0.85, 1.16)	0.86 (0.67, 1.11)
2005	1.05 (0.86, 1.28)	1.28 (0.99, 1.65)	0.79 (0.49, 1.28)	0.31 (0.21, 0.44)	1.06 (0.76, 1.48)	0.82 (0.6, 1.12)	0.98 (0.77, 1.25)	0.83 (0.58, 1.19)
2006	0.99 (0.67, 1.48)	1.46 (0.96, 2.21)	NA	0.29 (0.14, 0.6)	1.41 (0.85, 2.33)	0.66 (0.37, 1.19)	0.96 (0.7, 1.33)	NA
Regimen class								
NNRTI-EFV	1	1	1	1	1	1	1	1
NNRTI-NVP	1.56 (1.07, 2.27)	1.92 (1.09, 3.39)	1.13 (0.76, 1.66)	0.63 (0.46, 0.86)	5.92 (0.23, 150.8)^b^	2.57 (0.89, 7.41)	1.86 (1.29, 2.69)	1.66 (1.06, 2.62)
Non-NNRTI	2.02 (1.47, 2.77)	1.62 (1.07, 2.46)	2.9 (1.72, 4.87)	2.54 (1.78, 3.64)	1.57 (0.4, 6.06)	2.01 (1.21, 3.33)	1.5 (0.83, 2.71)	2.08 (1.74, 2.48)
ZDV Containing								
No ZDV	1	1	1	1	1	1	1	1
ZDV	1.02 (0.75, 1.39)	1.04 (0.69, 1.56)	0.7 (0.47, 1.05)	0.4 (0.29, 0.56)	5.92 (0.23, 152.2)^b^	1.26 (0.73, 2.17)	1.32 (0.99, 1.75)	1.14 (0.68, 1.91)

a Patients who started HAART before 2003 were included in the analysis, although we chose not to reference these earlier years in the table because at most sites there were very few patients.

b Hazard ratio estimates for ZDV and NNRTI-NVP for IHSS/HE-Honduras are erratic because of co-linearity: 219/220 patients on EFV were on ZDV, whereas 1/91 patients on NVP were on ZDV.

Multivariable analyses including HIV-1 RNA, hemoglobin, weight, and more refined regimen categories were performed for sites with sufficient data and are shown in Table S4 in the online supplemental material. Higher baseline weight was associated with a lower risk of changing regimens at IMTAvH-Peru. Higher baseline hemoglobin was predictive of a decreased risk of changing regimens at GHESKIO-Haiti. Patients treated with d4T had lower hemoglobin at baseline (medians of 9.7 vs. 11.0 mg/dl, p<0.0001). Higher baseline HIV-1 RNA was predictive of changing regimens at FA-Chile, but was not an independent predictor at FH-Argentina, INNSZ-Mexico, or IMTAvH-Peru.

The reported reasons for change during the first year are given in Table 6. Adverse events (AE) prompted change in 14.4% of HAART initiators, and were the most common reason for six of the seven sites. Other reasons for change were failure (1.3%), the availability of a better regimen or simplification (1.5%), drug supply problems (1.8%), and abandonment/adherence failures (1.1%). Of the patients who initiated HAART, 2.9% changed regimens for an undocumented reason.

**Table 6:** Reported reasons for changing initial HAART regimen in First Year.

	FH-Argentina	HUCFF-Brazil	FA-Chile	GHESKIO-Haiti	IHSS/HE-Honduras	INNSZ-Mexico	IMTAvH-Peru	Combined
	(n = 720)	(n = 522)	(n = 546)	(n = 1646)	(n = 324)	(n = 414)	(n = 854)	(n = 5026)
Changed Regimens								
(% of HAART initiators)	223 (31.0%)	151 (28.9%)	154 (28.2%)	324 (19.7%)	52 (16.0%)	77 (18.6%)	307 (35.9%)	1288 (25.6%)
Reasons for Changing								
(% of HAART initiators)								
Adverse Events	119 (16.5%)	79 (15.1%)	111 (20.3%)	133 (8.1%)	39 (12%)	40 (9.7%)	199 (23.3%)	720 (14.3%)
Types of Adverse Events a								
Hematology b	31 (4.3%)	25 (4.8%)	41 (7.5%)	70 (4.3%)	12 (3.7%)	22 (5.3%)	134 (15.7%)	335 (6.7%)
Skin	29 (4%)	9 (1.7%)	40 (7.3%)	20 (1.2%)	5 (1.5%)	0 (0%)	47 (5.5%)	150 (3%)
Intolerance	46 (6.4%)	18 (3.4%)	9 (1.6%)	7 (0.4%)	7 (2.2%)	5 (1.2%)	3 (0.4%)	95 (1.9%)
Nervous System, Central	6 (0.8%)	10 (1.9%)	10 (1.8%)	23 (1.4%)	10 (3.1%)	1 (0.2%)	8 (0.9%)	68 (1.4%)
Gynecomastia/Abnormal Fat Dist.	1 (0.1%)	1 (0.2%)	0 (0%)	16 (1%)	0 (0%)	0 (0%)	2 (0.2%)	20 (0.4%)
Liver	2 (0.3%)	4 (0.8%)	2 (0.4%)	0 (0%)	0 (0%)	0 (0%)	9 (1.1%)	17 (0.3%)
Nervous System, Peripheral	3 (0.4%)	2 (0.4%)	6 (1.1%)	2 (0.1%)	0 (0%)	0 (0%)	0 (0%)	13 (0.3%)
Kidney	0 (0%)	2 (0.4%)	0 (0%)	0 (0%)	0 (0%)	8 (1.9%)	0 (0%)	10 (0.2%)
Other Adverse Events c	2 (0.3%)	7 (1.3%)	3 (0.5%)	0 (0%)	1 (0.3%)	3 (0.7%)	1 (0.1%)	17 (0.3%)
Not Specified	2 (0.3%)	5 (1%)	1 (0.2%)	2 (0.1%)	7 (2.2%)	1 (0.2%)	0 (0%)	18 (0.4%)
Failure	5 (0.7%)	12 (2.3%)	2 (0.4%)	7 (0.4%)	0 (0%)	16 (3.9%)	22 (2.6%)	64 (1.3%)
Better Regimen Available/								
Simplification	5 (0.7%)	0 (0%)	13 (2.4%)	48 (2.9%)	1 (0.3%)	9 (2.2%)	0 (0%)	76 (1.5%)
Drug Supply Problems	2 (0.3%)	3 (0.6%)	0 (0%)	73 (4.4%)	1 (0.3%)	0 (0%)	10 (1.2%)	89 (1.8%)
Abandonment/Adherence Failure	11 (1.5%)	0 (0%)	16 (2.9%)	3 (0.2%)	3 (0.9%)	2 (0.5%)	19 (2.2%)	54 (1.1%)
Pregnancy-Related	5 (0.7%)	0 (0%)	0 (0%)	21 (1.3%)	1 (0.3%)	0 (0%)	0 (0%)	27 (0.5%)
Tuberculosis-Related	1 (0.1%)	2 (0.4%)	1 (0.2%)	23 (1.4%)	0 (0%)	2 (0.5%)	12 (1.4%)	41 (0.8%)
Other Reasons for Changing	20 (2.8%)	5 (1%)	10 (1.8%)	15 (0.9%)	3 (0.9%)	1 (0.2%)	27 (3.2%)	81 (1.6%)
Unknown	55 (7.6%)	50 (9.6%)	1 (0.2%)	1 (0.1%)	4 (1.2%)	7 (1.7%)	18 (2.1%)	136 (2.7%)

a Does not necessarily add to total number of adverse events because some patients experienced multiple events which prompted regimen change.

b Among those with a hematological adverse event, 73.4% were associated with anemia, 3.9% were not associated with anemia, and information regarding anemia was unspecified for the remaining 22.7%.

c Includes lactic acidosis (5), hypersensitivity reaction (2), and metabolic/dislipidemia (1).

The most common AE were hematological toxicity (6.7%), skin rash (3%) and gastrointestinal intolerance (1.9%), with substantial heterogeneity between sites. Of HAART initiators in IHSS/HE-Honduras, 3.7% changed regimens during the first year due to hematological adverse events, compared to 15.8% in IMTAvH-Peru. Among those with hematological adverse events, 73% were anemia, 4%. The distribution of HAART initiators changing due to skin rash also varied with FH-Argentina, FA-Chile and IMTAvH-Peru reporting 4.0%, 7.3%, and 5.5%, respectively, and other sites reporting <2%.

Within the first 3 months, AE were also the most common reported reason for changing regimens. Ten percent of patients changed regimens due to adverse events: 4.7% due to hematological toxicity and 2.8% due to skin rash. Forty-two patients from GHESKIO-Haiti who were on 3TC,ABC,ZDV switched to 3TC,ZDV,EFV in April/May of 2003 because this regimen became available and was deemed superior; each of these 42 patients had been on their initial regimen for less than 3 months.

For all NNRTI, boosted PI, and unboosted-PI-based regimens, AE were the main reason for change, although type of AE varied according to class. The most common AE for efavirenz-based regimens were hematological (5.7%), central nervous system (2.2%), and skin (1.6%); for nevirapine-based regimens: hematological (9.7%), skin (5.8%), liver (0.7%), and gastrointestinal intolerance (0.7%); for boosted PI-based regimens: gastrointestinal intolerance (8.3%), hematological (2.6%), and kidney (1.8%); and for unboosted PI- based regimens: gastrointestinal intolerance (7%), hematological (4.8%), and skin (1.1%).

Thirty patients died within 30 days of changing their initial HAART regimen. Adverse events were the reported reasons for change for 21 of these 30 patients. Most of these deaths were HIV-related or unspecified (tuberculosis 4, Kaposi's Sarcoma 2, wasting syndrome 2, Mycobacterium avium complex 1, Cryptococcosis 1, non-Hodgkin lymphoma 1, multiple opportunistic infections 1, AIDS-related but unspecified 1,); other causes included anemia 2, unspecified pulmonary infection 2, chronic renal failure 1, unspecified cancer 1, pancytopenia 1, and missing cause of death 10

## Discussion

This is the first multi-cohort study in Latin America and the Caribbean to describe rates of and reasons for changing initial HAART regimen. We found high rates of change early after treatment initiation with substantial variation across sites, ranging from 8–28% in the first 3 months and 18–41% in the first year. These rates are similar to those reported in other cohorts[10], [12]–[15], [35].

Also in agreement with other studies[10]–[14], [35], [36], adverse events were the main reason for change early after HAART initiation, with significant heterogeneity in the distribution of adverse events across sites. Hematological adverse events, >70% of which were anemia, were most common. This was most frequent in IMTAvH-Peru at 67%. Previous studies from Peru also reported anemia as a main reason for discontinuation (68%), and associated this finding with the use of standard 600 mg ZDV in low weight patients[21]. ZDV use was associated with an increase risk of discontinuation in the first 120 days of therapy and this early toxicity was associated with low baseline body weight. The high rates of HAART change due to anemia in IMTAvH-Peru may also be a reflection of baseline anemia and the fact that this site closely monitors anemia and changes HAART soon after its occurrence. The distribution of change in regimen due to skin rash also varied, with high rates in FH-Argentina, FA-Chile and IMTAvH-Peru and low rates elsewhere. This could be in part related to ethnicity, although this remains controversial[37]–[39].

GHESKIO-Haiti, with more advanced disease at baseline, had unexpectedly lower rates of change/discontinuation due to adverse events than other sites. However, the rates were similar when deaths were included as discontinuations. The availability of alternative drugs more than the occurrence of adverse events may explain this low rate.

As expected and previously reported[10], [12], patients were more likely to change therapy shortly after HAART initiation because of adverse events rather than treatment failure. Failure was given as the reason for change in 5% of changes, corresponding to 1.3% of HAART initiators. This low rate primarily may be explained by the short duration of follow-up.

Interruption in drug supply prompted changes in 2% of HAART initiators per site: its importance cannot be minimized since interruptions <30 days were ignored. Continuous provision of therapy is a key component of any successful HIV program, as treatment interruptions affect program effectiveness[5], [21].

Consistent with previous studies, individuals who died while on their first regimen were censored at the time of death in our primary analyses [10], [13], [14]. This analysis implicitly assumes that the frequency with which these individuals would have changed regimens had they continued to live is similar to the frequency of changing for those patients who remained in care. To examine the sensitivity of results to this assumption we performed additional analyses which categorized individuals who died while on their first regimen as having discontinued regimens. We also performed analyses, assuming those lost to follow-up stopped therapy. This latter assumption seems reasonable in sites where there were few other options for HIV care, but less reasonable for sites located in areas with several other points of care. Characteristics at HAART initiation of those subsequently lost to follow-up for the CCASAnet cohort have been described elsewhere[8]. Risk factors for changing/discontinuing first regimen were similar regardless of how those who died or were lost were classified

For the combined cohort, clinical AIDS prior to HAART initiation was identified as an independent predictor for treatment change. For FH-Argentina, in contrast to other sites, there was an increased risk of change at higher CD4 counts. A previous report suggested that patients with higher CD4 counts were at higher risk of GI intolerance whereas misclassification of gastrointestinal intolerance can occur in patients with low CD4 and associated opportunistic diseases[40]. Comorbidities in patients with advanced disease and concurrent treatments for opportunistic diseases may affect antiretroviral tolerance and thereby increase risk of toxicities. Late HAART initiation was associated with higher rates of treatment change. Approximately 50% of the patients in the combined cohort started therapy at less than 100 CD4/mL, highlighting the urgent need for timely diagnosis and treatment of HIV-positive patients.

Efavirenz-based regimens had the lowest hazard for change. The increased hazard for change of NVP-based regimens was especially pronounced in IMTAvH-Peru. This may be explained by the use of fixed dose combinations containing ZDV and anemia frequency. In spite of the high proportions of hematological toxicity, the hazards of change for ZDV- and non-ZDV-containing regimens were similar. We believe that since patients with anemia at baseline were typically assigned to non-ZDV containing regimens (primarily d4T), ZDV-treated patients were “protected” from subsequent change due to anemia. However, baseline anemia status was not consistently collected for all sites.

We failed to identify consistent associations between gender or age and risk of antiretroviral change, although other studies have found that younger age and female gender predict change[13].

After the first change, most patients remained on regimens within the same class. The outcome of second regimens was not assessed as it was beyond the scope of this study.

Our study had several limitations. Adverse events or regimen failures were computed only if they prompted regimen change. Therefore, their frequency cannot be used to estimate their occurrence, but rather the frequency of events deemed significant enough to prompt a regimen change. Strategies for changing regimens varied throughout the region and have evolved over time. Therefore, differences between sites regarding rates of toxicities, for example, may reflect site differences in guidelines for changing regimens or availability of alternative regimens. In addition, these data were collected retrospectively, and differences between sites in reasons for change and adverse events may reflect varying levels of data capture. Results of pooled analyses should be treated with caution given the heterogeneity seen in our cohort

We did not consider treatment modifications or interruptions shorter than 30 days because registration of such events varied across sites and shorter discontinuations were less likely to have been recorded. We recognize the potential impact of minor interruptions on treatment outcomes, particularly using NNRTI-based regimens; such interruptions occur frequently in real life but generally have not been considered in other cohort studies[14], [41].

Although we controlled for key variables such as CD4 count and clinical stage, patients were not randomly assigned to their initial regimens, so rates of change may be higher for certain regimens due to baseline characteristics rather than the regimen itself. However, most of the associations observed in this study are similar to those reported elsewhere[10], [12]–[16], [35], [39].

In conclusion, the high rate of change due to adverse events is consistent with studies from other cohorts. Heterogeneity between sites may be explained by differences in baseline characteristics at HAART initiation, programmatic differences, demographics and population genetics. Unfortunately, with only 7-sites, we are unable to perform analyses to investigate the impact of site-specific factors on regimen change. Efavirenz-based regimens were widely used and showed a lower rate of discontinuation compared to nevirapine or PI-based treatments.

## Supporting Information

Table S1 Adjusted Hazard Ratios (95% Confidence Intervals) for Regimen Change in First Year by most Common Regimens.
(0.04 MB DOC)

Table S2 Adjusted Hazard Ratios (95% Confidence Intervals) for Regimen Change/Discontinuation in First Year counting Death as a Discontinuation.
(0.06 MB DOC)

Table S3 Adjusted Hazard Ratios (95% Confidence Intervals) for Regimen Change/Discontinuation in First Year, counting Death and Loss to Follow-up as Discontinuations.
(0.06 MB DOC)

Table S4 Adjusted Hazard Ratios (95% Confidence Intervals) for Regimen Change/Discontinuation in the First Year including available Predictors for each Site.
(0.09 MB DOC)
