# Supplementary material for: Rates and Reasons for Early Change of First HAART in HIV-1-Infected Patients in 7 Sites throughout the Caribbean and Latin America
Source: PLoS One. 2010 Jun 1;5(6):e10490. doi: 10.1371/journal.pone.0010490 (PMC2879360; doi:10.1371/journal.pone.0010490)
Supplement: Table S1 — Adjusted Hazard Ratios (95% Confidence Intervals) for Regimen Change in First Year by most Common Regimens. (0.04 MB DOC) [file pone.0010490.s001.doc]

**Table S1.** Adjusted Hazard Ratios (95% Confidence Intervals) for Regimen Change in First Year by most Common Regimens

|  | FH-Argentina | HUCFF-Brazil | FA-Chile | GHESKIO-Haiti | IHSS/HE-Honduras | INNSZ-Mexico | IMTAvH-Peru |
| --- | --- | --- | --- | --- | --- | --- | --- |
| 3TC,ZDV,EFV | 1 | 1 | 1 | 1 | 1 | 1 | 1 |
| 3TC,ZDV,NVP | 1.51 (1,2.29) | 2.26 (1.19,4.32) | 1.07 (0.7,1.65) | 0.53 (0.38,0.75) | NA ^b^ | 4.17 (1.42,12.21) | 1.79 (1.18,2.71) |
| 3TC,D4T,NVP | 1.8 (0.77,4.23) | 2.47 (0.75,8.09) | 1.17 (0.27,5.09) | 3.05 (1.82,5.12) | 0.97 (0.49,1.91) | NA | 1.41 (0.84,2.36) |
| 3TC,D4T,EFV | 1.29 (0.72,2.3) | 1.07 (0.48,2.41) | 1.34 (0.77,2.33) | 3.13 (1.91,5.14) | NA | 0.72 (0.22,2.4) | 1.22 (0.46,3.2) |
| 3TC,ABC,ZDV | 2.14 (1.19,3.88) | 1.62 (0.72,3.62) | 3.45 (1.43,8.34) | 5.48 (3.69,8.13) | NA | 5.51 (1.18,25.78) | 2.44 (0.31,19.08) |
| Other ^a^ | 2.03 (1.43,2.87) | 1.83 (1.18,2.85) | 1.68 (1.05,2.7) | 1.26 (0.67,2.37) | 2.13 (0.74,6.09) | 1.7 (0.99,2.91) | 1.2 (0.74,1.97) |

^a^ There were 94 other regimens.

^b^ NA: Not available because too few people started the regimen.

^c^ Analyses were adjusted for sex, age, AIDS, CD4, and year of HAART initiation. Hazard ratios for these covariates were similar to those presented in Table 3 of the main text.
