# Supplementary material for: Rates and Reasons for Early Change of First HAART in HIV-1-Infected Patients in 7 Sites throughout the Caribbean and Latin America
Source: PLoS One. 2010 Jun 1;5(6):e10490. doi: 10.1371/journal.pone.0010490 (PMC2879360; doi:10.1371/journal.pone.0010490)
Supplement: Table S2 — Adjusted Hazard Ratios (95% Confidence Intervals) for Regimen Change/Discontinuation in First Year counting Death as a Discontinuation. (0.06 MB DOC) [file pone.0010490.s002.doc]

**Table S2.** Adjusted Hazard Ratios (95% Confidence Intervals) for Regimen Change/Discontinuation in First Year counting
Death as a Discontinuation.

|  | FH-Argentina | HUCFF-Brazil | FA-Chile | GHESKIO-Haiti | IHSS/HE-Honduras | INNSZ-Mexico | IMTAvH-Peru | Combined |
| --- | --- | --- | --- | --- | --- | --- | --- | --- |
|  |  |  |  |  |  |  |  |  |
| Male | 0.74 (0.56, 0.99) | 1.12 (0.79, 1.58) | 0.72 (0.47, 1.11) | 0.87 (0.7, 1.09) | 1.22 (0.76, 1.94) | 1.11 (0.57, 2.16) | 1.01 (0.8, 1.29) | 0.97 (0.82, 1.15) |
|  |  |  |  |  |  |  |  |  |
| Age (per 10 years) | 1.04 (0.9, 1.19) | 1.01 (0.87, 1.18) | 0.97 (0.82, 1.14) | 0.98 (0.89, 1.08) | 1.09 (0.85, 1.38) | 0.93 (0.74, 1.16) | 0.97 (0.87, 1.09) | 1 (0.95, 1.05) |
|  |  |  |  |  |  |  |  |  |
| AIDS | 1.64 (1.21, 2.22) | 1.24 (0.81, 1.89) | 1.2 (0.83, 1.75) | 1.48 (1.23, 1.79) | 1.12 (0.71, 1.76) | 1.59 (0.96, 2.65) | 1.41 (1.1, 1.8) | 1.44 (1.28, 1.61) |
|  |  |  |  |  |  |  |  |  |
| CD4 count (cells/mL) |  |  |  |  |  |  |  |  |
| 100 vs. 50 | 1.11 (1.03, 1.19) | 0.88 (0.8, 0.97) | 0.97 (0.87, 1.09) | 0.9 (0.85, 0.96) | 0.93 (0.79, 1.09) | 0.92 (0.79, 1.08) | 0.85 (0.79, 0.91) | 0.93 (0.86, 1.01) |
| 200 vs. 50 | 1.29 (1.08, 1.53) | 0.73 (0.58, 0.92) | 0.93 (0.71, 1.22) | 0.78 (0.68, 0.9) | 0.83 (0.56, 1.23) | 0.83 (0.57, 1.2) | 0.67 (0.57, 0.79) | 0.85 (0.7, 1.02) |
| 350 vs. 50 | 1.51 (1.14, 2.01) | 0.6 (0.41, 0.87) | 0.89 (0.57, 1.39) | 0.67 (0.53, 0.84) | 0.74 (0.39, 1.4) | 0.73 (0.39, 1.35) | 0.52 (0.39, 0.68) | 0.76 (0.56, 1.04) |
|  |  |  |  |  |  |  |  |  |
| Year of HAART initiation |  |  |  |  |  |  |  |  |
| 2003 (ref) | 1 | 1 | 1 | 1 | 1 | 1 | 1 | 1 |
| 2004 | 1.05 (0.95, 1.17) | 1.13 (1.01, 1.26) | 0.97 (0.83, 1.13) | 0.37 (0.3, 0.45) | 0.96 (0.78, 1.18) | 0.98 (0.79, 1.22) | 1.07 (0.91, 1.25) | 0.88 (0.67, 1.16) |
| 2005 | 1.04 (0.86, 1.26) | 1.28 (1, 1.65) | 0.77 (0.49, 1.23) | 0.36 (0.27, 0.47) | 1.05 (0.78, 1.41) | 0.88 (0.66, 1.19) | 1.1 (0.85, 1.41) | 0.86 (0.6, 1.25) |
| 2006 | 0.99 (0.67, 1.46) | 1.47 (0.98, 2.21) | NA | 0.38 (0.22, 0.65) | 1.28 (0.84, 1.95) | 0.74 (0.43, 1.25) | 1.02 (0.75, 1.4) | NA |
|  |  |  |  |  |  |  |  |  |
| Regimen class |  |  |  |  |  |  |  |  |
| NNRTI-EFV | 1 | 1 | 1 | 1 | 1 | 1 | 1 | 1 |
| NNRTI-NVP | 1.55 (1.06, 2.24) | 1.87 (1.06, 3.3) | 1.08 (0.75, 1.56) | 0.77 (0.6, 0.99) | 4.93 (0.36, 67.55) | 2.35 (0.82, 6.72) | 1.83 (1.3, 2.57) | 1.68 (1.09, 2.57) |
| Non-NNRTI | 2.01 (1.47, 2.74) | 1.67 (1.11, 2.51) | 2.54 (1.55, 4.19) | 1.9 (1.38, 2.62) | 1.22 (0.34, 4.46) | 2.01 (1.26, 3.21) | 1.52 (0.87, 2.64) | 1.92 (1.64, 2.25) |
|  |  |  |  |  |  |  |  |  |
| ZDV Containing |  |  |  |  |  |  |  |  |
| No ZDV | 1 | 1 | 1 | 1 | 1 | 1 | 1 | 1 |
| ZDV | 1 (0.74, 1.36) | 0.99 (0.67, 1.46) | 0.65 (0.45, 0.94) | 0.39 (0.3, 0.51) | 3.92 (0.28, 54.29) | 1.11 (0.68, 1.81) | 1.09 (0.85, 1.41) | 1.03 (0.59, 1.79) |
