# Supplementary material for: Rates and Reasons for Early Change of First HAART in HIV-1-Infected Patients in 7 Sites throughout the Caribbean and Latin America
Source: PLoS One. 2010 Jun 1;5(6):e10490. doi: 10.1371/journal.pone.0010490 (PMC2879360; doi:10.1371/journal.pone.0010490)
Supplement: Table S3 — Adjusted Hazard Ratios (95% Confidence Intervals) for Regimen Change/Discontinuation in First Year, counting Death and Loss to Follow-up as Discontinuations. (0.06 MB DOC) [file pone.0010490.s003.doc]

**Table S3.** Adjusted Hazard Ratios (95% Confidence Intervals) for Regimen Change/Discontinuation in First Year, counting Death and Loss to Follow-up as Discontinuations

|  | FH-Argentina | HUCFF-Brazil | FA-Chile | GHESKIO-Haiti | IHSS/HE-Honduras | INNSZ-Mexico | IMTAvH-Peru | Combined |
| --- | --- | --- | --- | --- | --- | --- | --- | --- |
|  |  |  |  |  |  |  |  |  |
| Male | 0.77 (0.59, 1) | 1.11 (0.79, 1.54) | 0.66 (0.44, 0.99) | 0.84 (0.68, 1.03) | 1.24 (0.77, 1.97) | 1.05 (0.57, 1.94) | 1.01 (0.8, 1.27) | 0.95 (0.78, 1.15) |
|  |  |  |  |  |  |  |  |  |
| Age (per 10 years) | 0.99 (0.87, 1.12) | 1.02 (0.88, 1.18) | 0.95 (0.81, 1.12) | 0.99 (0.9, 1.09) | 1.1 (0.86, 1.39) | 0.92 (0.75, 1.13) | 0.97 (0.87, 1.08) | 0.99 (0.95, 1.04) |
|  |  |  |  |  |  |  |  |  |
| AIDS | 1.54 (1.17, 2.03) | 1.13 (0.76, 1.68) | 1.14 (0.79, 1.66) | 1.43 (1.2, 1.71) | 1.09 (0.7, 1.72) | 1.51 (0.94, 2.42) | 1.44 (1.13, 1.82) | 1.39 (1.25, 1.55) |
|  |  |  |  |  |  |  |  |  |
| CD4 count (cells/mL) |  |  |  |  |  |  |  |  |
| 100 vs. 50 | 1.1 (1.03, 1.18) | 0.88 (0.8, 0.96) | 0.93 (0.84, 1.05) | 0.91 (0.86, 0.96) | 0.93 (0.79, 1.09) | 0.93 (0.81, 1.07) | 0.88 (0.82, 0.94) | 0.94 (0.87, 1.01) |
| 200 vs. 50 | 1.26 (1.08, 1.48) | 0.73 (0.59, 0.91) | 0.85 (0.65, 1.11) | 0.8 (0.7, 0.92) | 0.84 (0.57, 1.23) | 0.85 (0.6, 1.19) | 0.73 (0.62, 0.85) | 0.86 (0.72, 1.02) |
| 350 vs. 50 | 1.47 (1.13, 1.91) | 0.6 (0.42, 0.86) | 0.77 (0.49, 1.19) | 0.7 (0.56, 0.87) | 0.75 (0.4, 1.4) | 0.76 (0.43, 1.33) | 0.59 (0.45, 0.77) | 0.77 (0.58, 1.03) |
|  |  |  |  |  |  |  |  |  |
| Year of HAART initiation |  |  |  |  |  |  |  |  |
| 2003 (ref) | 1 | 1 | 1 | 1 | 1 | 1 | 1 | 1 |
| 2004 | 1.1 (0.99, 1.21) | 1.15 (1.04, 1.28) | 1.02 (0.88, 1.19) | 0.4 (0.33, 0.49) | 0.96 (0.78, 1.17) | 1.04 (0.85, 1.28) | 1.1 (0.94, 1.29) | 0.92 (0.71, 1.19) |
| 2005 | 1.1 (0.92, 1.31) | 1.35 (1.06, 1.72) | 0.89 (0.57, 1.38) | 0.42 (0.32, 0.54) | 1.04 (0.77, 1.4) | 0.95 (0.72, 1.26) | 1.16 (0.9, 1.5) | 0.93 (0.66, 1.31) |
| 2006 | 1.05 (0.74, 1.49) | 1.59 (1.08, 2.34) | NA | 0.47 (0.29, 0.77) | 1.28 (0.85, 1.94) | 0.78 (0.48, 1.26) | 1.08 (0.79, 1.47) | NA |
|  |  |  |  |  |  |  |  |  |
| Regimen class |  |  |  |  |  |  |  |  |
| NNRTI-EFV | 1 | 1 | 1 | 1 | 1 | 1 | 1 | 1 |
| NNRTI-NVP | 1.55 (1.1, 2.17) | 1.97 (1.15, 3.36) | 1.08 (0.75, 1.55) | 0.81 (0.64, 1.02) | 4.93 (0.36, 68.17) | 2.31 (0.9, 5.92) | 1.74 (1.26, 2.42) | 1.67 (1.11, 2.49) |
| Non-NNRTI | 2.11 (1.59, 2.79) | 1.68 (1.13, 2.49) | 2.64 (1.62, 4.3) | 1.82 (1.33, 2.5) | 1.19 (0.33, 4.29) | 1.85 (1.2, 2.85) | 1.44 (0.84, 2.47) | 1.91 (1.64, 2.22) |
|  |  |  |  |  |  |  |  |  |
| ZDV Containing |  |  |  |  |  |  |  |  |
| No ZDV | 1 | 1 | 1 | 1 | 1 | 1 | 1 | 1 |
| ZDV | 0.99 (0.76, 1.31) | 0.88 (0.61, 1.28) | 0.68 (0.48, 0.97) | 0.4 (0.31, 0.51) | 3.77 (0.27, 52.61) | 0.94 (0.6, 1.45) | 1.16 (0.9, 1.49) | 0.99 (0.61, 1.62) |
