# Supplementary material for: Rates and Reasons for Early Change of First HAART in HIV-1-Infected Patients in 7 Sites throughout the Caribbean and Latin America
Source: PLoS One. 2010 Jun 1;5(6):e10490. doi: 10.1371/journal.pone.0010490 (PMC2879360; doi:10.1371/journal.pone.0010490)
Supplement: Table S4 — Adjusted Hazard Ratios (95% Confidence Intervals) for Regimen Change/Discontinuation in the First Year including available Predictors for each Site. (0.09 MB DOC) [file pone.0010490.s004.doc]

**Table S4.** Adjusted Hazard Ratios (95% Confidence Intervals) for Regimen Change/Discontinuation in the First Year including available Predictors for each Site

|  | FH-Argentina | HUCFF-Brazil | FA-Chile | GHESKIO-Haiti | IHSS/HE-Honduras | INNSZ-Mexico | IMTAvH-Peru |
| --- | --- | --- | --- | --- | --- | --- | --- |
|  |  |  |  |  |  |  |  |
| Male | 0.73 (0.55, 0.97) | 1.06 (0.75, 1.51) | 0.74 (0.46, 1.18) | 0.81 (0.62, 1.05) | 0.93 (0.51, 1.71) | 1.16 (0.56, 2.39) | 1.31 (0.99, 1.73) |
|  |  |  |  |  |  |  |  |
| Age (per 10 years) | 1.02 (0.89, 1.18) | 1 (0.85, 1.17) | 0.93 (0.78, 1.11) | 0.91 (0.8, 1.03) | 1 (0.73, 1.36) | 0.89 (0.69, 1.15) | 0.99 (0.88, 1.11) |
|  |  |  |  |  |  |  |  |
| Clinical AIDS | 1.64 (1.2, 2.25) | 1.3 (0.84, 2) | 1.14 (0.77, 1.71) | 1.08 (0.85, 1.37) | 0.86 (0.46, 1.61) | 1.29 (0.72, 2.31) | 1.17 (0.9, 1.5) |
|  |  |  |  |  |  |  |  |
| CD4 count (cells/mL) |  |  |  |  |  |  |  |
| 100 vs. 50 | 1.13 (1.05, 1.22) | 0.9 (0.82, 0.99) | 1.04 (0.92, 1.17) | 0.97 (0.9, 1.04) | 0.95 (0.77, 1.18) | 0.94 (0.79, 1.1) | 0.96 (0.89, 1.04) |
| 200 vs. 50 | 1.34 (1.11, 1.62) | 0.78 (0.62, 0.98) | 1.09 (0.81, 1.46) | 0.93 (0.78, 1.09) | 0.89 (0.54, 1.48) | 0.85 (0.57, 1.27) | 0.91 (0.75, 1.1) |
| 350 vs. 50 | 1.62 (1.19, 2.21) | 0.67 (0.46, 0.97) | 1.15 (0.7, 1.87) | 0.88 (0.67, 1.16) | 0.83 (0.36, 1.9) | 0.77 (0.4, 1.49) | 0.85 (0.62, 1.17) |
|  |  |  |  |  |  |  |  |
| Year of HAART Initiation |  |  |  |  |  |  |  |
| 2003 (ref) | 1 | 1 | 1 | 1 | 1 | 1 | 1 |
| 2004 | 1.07 (0.97, 1.17) | 1.12 (1, 1.26) | 0.98 (0.83, 1.16) | 0.37 (0.29, 0.47) | 0.94 (0.75, 1.18) | 0.99 (0.76, 1.3) | 0.99 (0.89, 1.11) |
| 2005 | 1.14 (0.95, 1.37) | 1.27 (0.97, 1.65) | 0.78 (0.48, 1.27) | 0.28 (0.19, 0.41) | 1.05 (0.74, 1.48) | 0.86 (0.6, 1.24) | 0.99 (0.79, 1.23) |
| 2006 | 1.21 (0.92, 1.6) | 1.44 (0.95, 2.21) | NA | 0.23 (0.11, 0.48) | 1.38 (0.83, 2.31) | 0.67 (0.37, 1.24) | 0.98 (0.71, 1.37) |
|  |  |  |  |  |  |  |  |
| HIV-1 RNA (per log_10_) | 1.07 (0.91, 1.25) | NA | 1.38 (1.06, 1.8) | NA | NA | 0.9 (0.53, 1.53) | 1.09 (0.93, 1.27) |
|  |  |  |  |  |  |  |  |
| Weight (per 10 kg) | NA | NA | NA | 0.99 (0.89, 1.1) | 1.04 (0.78, 1.4) | 0.89 (0.72, 1.09) | 0.78 (0.69, 0.88) |
|  |  |  |  |  |  |  |  |
| Hemoglobin (per g/dL) | NA | NA | NA | 0.87 (0.82, 0.93) | 1.01 (0.86, 1.19) | 0.97 (0.87, 1.07) | NA |
|  |  |  |  |  |  |  |  |
| Injection Drug Use | 1.09 (0.73, 1.61) | NA | NA | NA | NA | NA | NA |
|  |  |  |  |  |  |  |  |
| Regimen class |  |  |  |  |  |  |  |
| NNRTI-EFV | 1 | 1 | 1 | 1 | 1 | 1 | 1 |
| NNRTI-NVP | 1.62 (1.11, 2.36) | 1.86 (1.05, 3.3) | 1.26 (0.85, 1.87) | 0.58 (0.43, 0.79) | 5.76 (0.21, 157.2) | 2.72 (0.93, 7.92) | 1.9 (1.34, 2.7) |
| Boosted PI | 1.93 (1.36, 2.72) | 1.75 (1, 3.06) | NA | NA | NA | 1.82 (1.06, 3.14) | 1.37 (0.65, 2.89) |
| PI | 3.2 (1.58, 6.45) | 1.64 (0.95, 2.84) | 2.63 (1.36, 5.08) | NA | NA | NA | 1.85 (0.77, 4.44) |
| Other | 2.06 (1.22, 3.48) | 1.41 (0.63, 3.13) | NA | 5.25 (3.54, 7.78) | NA | NA | 2.24 (0.64, 7.76) |
| Boosted PI / PI | NA | NA | NA | 0.37 (0.13, 1.03) | NA | NA | NA |
| Boosted PI / Other | NA | NA | 3.61 (1.75, 7.45) | NA | NA | NA | NA |
| PI / Other | NA | NA | NA | NA | NA | 3.25 (1.32, 8.01) | NA |
| Boosted PI / PI / Other | NA | NA | NA | NA | 1.52 (0.38, 6.06) | NA | NA |
|  |  |  |  |  |  |  |  |
| ZDV Containing |  |  |  |  |  |  |  |
| No ZDV | 1 | 1 | 1 | 1 | 1 | 1 | 1 |
| ZDV | 1.07 (0.97, 1.17) | 1.12 (1, 1.26) | 0.72 (0.48, 1.07) | 0.39 (0.27, 0.56) | 5.74 (0.21, 157.5) | 1.3 (0.75, 2.26) | 1.4 (1.05, 1.86) |

NA: Not available because of missing data.
